# Cerebellar Metastasis From Radioiodine-Refractory Widely Invasive Oncocytic (Hurthle Cell) Carcinoma of the Thyroid in a Young Adult

**DOI:** 10.7759/cureus.117612

**Published:** 2026-10-07

**Authors:** Meera Davuluri, Maansi Bhasin, Ahmed Cheema, James Miller

**Affiliations:** 1 Internal Medicine, Froedtert Hospital, Kenosha, USA; 2 Pulmonology, Rosalind Franklin University of Medicine and Science, North Chicago, USA; 3 Neurosurgery, Froedtert Hospital, Milwaukee, USA; 4 Pathology, Froedtert Hospital, Milwaukee, USA

**Keywords:** biochemical incomplete response, brain metastasis, cerebellar metastasis, fdg pet/ct, hurthle cell carcinoma, oncocytic thyroid carcinoma, thyroglobulin surveillance, total thyroidectomy

## Abstract

Oncocytic (Hurthle cell) carcinoma is an uncommon thyroid malignancy that tends to spread through the bloodstream and often concentrates radioactive iodine poorly. Spread to the brain is rare, and a solitary cerebellar deposit is rarer still. We describe a 44-year-old man who presented with neck swelling and was found to have a widely invasive oncocytic carcinoma with extensive vascular invasion and a minor classic papillary component (5%). He underwent total thyroidectomy with central neck dissection followed by radioactive iodine ablation with 150 mCi of iodine-131. The post-therapy scan showed uptake confined to the thyroid bed. Over the next 18 months, his serum thyroglobulin climbed steadily from 1.5 µg/L to 11.6 µg/L even though the thyroid-stimulating hormone (TSH)-stimulated diagnostic whole-body iodine scan remained negative. Fluorodeoxyglucose positron emission tomography/computed tomography then showed multiple hypermetabolic bilateral pulmonary nodules suspicious for metastases. Roughly 22 months after thyroidectomy, he developed progressive dizziness and headache. Magnetic resonance imaging (MRI) revealed a 3.3 × 3.2 × 3.1 cm mixed cystic and solid left cerebellar mass with surrounding vasogenic edema and mass effect on the brainstem. The mass was resected through a left suboccipital craniotomy. Histology showed a malignant epithelioid neoplasm with nested and trabecular growth, abundant granular eosinophilic cytoplasm, prominent nucleoli, scattered bizarre giant nuclei, and 8 mitoses per 2 mm^2^ without necrosis. The tumor cells expressed cytokeratin AE1/AE3, CK7, TTF-1, and PAX-8 and were negative for CK20, CDX-2, synaptophysin, chromogranin, and S100, confirming thyroid origin. The metastasis fulfilled criteria for high-grade differentiated thyroid carcinoma with oncocytic features. His symptoms resolved after surgery, and stereotactic radiosurgery for the resection cavity was recommended. A rising thyroglobulin with negative iodine imaging represents a biochemical incomplete response and should prompt a search for structural disease recurrence. Because the brain lesion in our patient came to light only when symptoms appeared, brain MRI may be worth considering in patients with radiodiodine-refractory distant metastases.

## Introduction

Oncocytic carcinoma of the thyroid, long known as Hurthle cell carcinoma, is treated in the 2022 World Health Organization classification as a malignancy in its own right rather than a variant of follicular carcinoma, defined by a population of at least 75% oncocytic cells packed with mitochondria [1]. Compared with conventional follicular carcinoma, it invades vessels more often, disseminates hematogenously, and responds poorly to radioactive iodine (RAI). Sequencing work has offered an explanation: these tumors carry near-homoplasmic mitochondrial complex I mutations and exhibit such widespread chromosomal loss that many approach a haploid genome, features that persist in metastases and correlate with both radioiodine resistance and strong fluorodeoxyglucose (FDG) uptake [2,3].

The lungs and skeleton are the usual sites of distant spread. Brain involvement is seen in only about 1% of differentiated thyroid cancers and carries a poor outlook, although resection prolongs survival in well-selected patients, and modern series favor surgery or stereotactic radiosurgery over whole-brain radiotherapy [4,5].

Here we report the case of a 44-year-old man with widely invasive oncocytic carcinoma who developed a solitary, symptomatic cerebellar metastasis roughly 22 months after thyroidectomy and RAI ablation. His course, from a quietly rising thyroglobulin through negative iodine scans to histologically proven intracranial disease, traces the diagnostic pathway that clinicians face when thyroglobulin rises despite negative radioiodine imaging.

## Case presentation

A 44-year-old man presented in August 2023 with painless anterior neck swelling. He was euthyroid (free thyroxine 13.8 pmol/L; thyroid-stimulating hormone (TSH) 2.10 mIU/L). Neck ultrasound showed an enlarged right lobe measuring 5.9 × 4.2 × 4.6 cm, containing a dominant 5.4 × 3.9 cm solid nodule graded TI-RADS 5 under the American College of Radiology system, along with a 2 × 1 cm left lobe nodule graded TI-RADS 4; no suspicious cervical nodes were seen [6]. Fine-needle aspiration of both nodules was performed; the right nodule was reported as a Hurthle cell (oncocytic) neoplasm (Bethesda category IV) and the left nodule as atypia of undetermined significance (Bethesda category III) [7]. The preoperative serum thyroglobulin was strikingly high at 1,016 µg/L, with a thyroglobulin antibody (TgAb) titer of 11 IU/mL.

Total thyroidectomy with central compartment dissection followed in September 2023. The tumor measured 7 × 5 × 3 cm. Pathology showed a widely invasive oncocytic (Hurthle cell) carcinoma making up 95% of the tumor, with extensive vascular invasion, alongside a minor (5%) component of classic papillary carcinoma. The report described this as a single mixed carcinoma in the right lobe, which was almost replaced by a multifocal, multinodular tumor and did not state whether the papillary component was intermixed or formed a separate focus. The left lobe nodule showed benign oncocytic nodular disease; nine of 26 dissected nodes contained metastases attributable to the papillary component. The thyroidectomy was performed abroad, and the slides were not available for review; the outside report described focal tumor necrosis (about 3%) in the oncocytic component, together with lymphatic and perineural invasion, but did not report a mitotic count. There was no extrathyroidal extension, and all margins were negative, the closest being under 1 mm. The tumor was staged as pT3 pN1a under the eighth edition of the American Joint Committee on Cancer system [8]. No distant metastases were evident at initial staging, which corresponds to stage I in a patient younger than 55 years. In November 2023, on levothyroxine with a TSH of 2.01 mIU/L, the postoperative thyroglobulin had fallen to 1.54 µg/L. In December 2023, after TSH stimulation to 93.7 mIU/L (stimulated thyroglobulin 1.48 µg/L; TgAb 10 IU/mL), he received 150 mCi of iodine-131. The post-therapy single-photon emission computed tomography/computed tomography (SPECT/CT) showed good uptake in the thyroid bed and nothing elsewhere. The full clinical course and serial laboratory values are laid out in Table 1.

**Table 1:** Timeline of clinical events, investigations, and serial laboratory values. Reference ranges: TgAb 0-100 IU/mL; TSH 0.27-4.2 mIU/L; on thyroid hormone after total thyroidectomy and ablation, the target thyroglobulin is undetectable. T4: thyroxine; TSH: thyroid-stimulating hormone; ACR TI-RADS: American College of Radiology Thyroid Imaging Reporting and Data System; TgAb: thyroglobulin antibody; I-131: iodine-131; SPECT/CT: single-photon emission computed tomography/computed tomography; CT: computed tomography; FDG-PET/CT: fluorodeoxyglucose positron emission tomography/computed tomography; SUVmax: maximum standardized uptake value; MRI: magnetic resonance imaging.

Time point	Event/investigation	Key findings
August 2023	Presentation with neck swelling; thyroid function tests	Free T4 13.8 pmol/L; TSH 2.10 mIU/L (euthyroid)
August 2023	Neck ultrasound	Right lobe 5.9 × 4.2 × 4.6 cm with dominant 5.4 × 3.9 cm nodule (ACR TI-RADS 5); left lobe 2 × 1 cm nodule (ACR TI-RADS 4)
August 2023	Fine-needle aspiration of both nodules	Right Hurthle cell (oncocytic neoplasm), left: atypia of undetermined significance
September 2023	Preoperative laboratory studies	Thyroglobulin 1,016 µg/L (elevated); TgAb 11 IU/mL; TSH 2.07 mIU/L; calcium 9.42 mg/dL; creatinine 1.54 mg/dL
September 2023	Total thyroidectomy with central neck dissection	Widely invasive oncocytic (Hurthle cell) carcinoma (95%) with extensive vascular invasion, focal tumor necrosis (about 3%), and a minor classic papillary carcinoma component (5%); 9 of 26 lymph nodes positive (papillary component); pT3 pN1a
November 2023	Postoperative surveillance and neck ultrasound	Thyroglobulin 1.54 µg/L; TgAb 11 IU/mL; TSH 2.01 mIU/L; neck ultrasound: 7-mm soft tissue in the right thyroid bed, no suspicious nodes
December 2023	TSH-stimulated labs and radioactive iodine ablation (150 mCi I-131)	TSH 93.7 mIU/L, thyroglobulin 1.48 µg/L, and TgAb 10 IU/mL at stimulation; post-therapy SPECT/CT showed uptake in the thyroid bed only, with no distant iodine-avid disease
May 2024	Surveillance and neck ultrasound	Thyroglobulin 1.56 µg/L; TSH 3.96 mIU/L; neck ultrasound: small echogenic tissue in the right thyroid bed (residual tissue or fibrosis), reactive-appearing cervical nodes
September 2024	Surveillance and neck ultrasound	Thyroglobulin 3.21 µg/L (rising); TSH 0.29 mIU/L; neck ultrasound unchanged
January 2025	Surveillance and TSH-stimulated whole-body scan (5.06 mCi I-131)	Thyroglobulin 5.34 µg/L (TSH 0.01 mIU/L), then 7.34 µg/L (TSH-stimulated, TSH 134 mIU/L); the whole-body scan showed resolved thyroid-bed uptake and no iodine-avid lesions
May 2025	Surveillance and FDG-PET/CT (Vertex to mid-thigh)	Thyroglobulin 11.6 µg/L; PET showed multiple bilateral pulmonary nodules suspicious for metastasis (largest 7 mm, SUVmax 8.9) and a right hilar focus (SUVmax 3.8); TSH 0.2 mIU/L; brain not reliably assessable because of physiological uptake
July 2025	Emergency department presentation with progressive dizziness and headache; chest CT on admission	Multiple bilateral pulmonary nodules, radiologically suspicious for metastasis, again seen on chest CT
July 2025	Brain MRI with and without contrast	Mixed cystic and solid left cerebellar lesion, 3.3 × 3.2 × 3.1 cm overall (solid component 2.5 × 2.2 × 2.8 cm), with vasogenic edema, mild intralesional hemorrhage, and mass effect on the brainstem; intravenous dexamethasone started
July 2025	Left suboccipital craniotomy and resection	Metastatic carcinoma of thyroid origin confirmed on histopathology and immunohistochemistry; 8 mitoses per 2 mm^2^, no necrosis; high-grade differentiated thyroid carcinoma with oncocytic features
July 2025	Postoperative course	Neurological symptoms resolved; stereotactic radiosurgery to the resection cavity recommended, to be delivered overseas (as the patient was working overseas); no further follow-up available

On surveillance, the thyroglobulin did not stay down. It rose from 1.56 µg/L in May 2024 to 3.21 µg/L that September and 5.34 µg/L in January 2025, with TgAb stable at low titer. Neck ultrasound in May and September 2024 showed a small, unchanged focus of echogenic soft tissue in the right thyroid bed, thought to be residual tissue or fibrosis, and a few reactive-appearing upper cervical lymph nodes, without suspicious nodes. On levothyroixine, his TSH was 2.01 mIU/L in November 2023 and 3.96 mIU/L in May 2024 and lower thereafter (0.29 mIU/L in September 2024, 0.01 mIU/L in January 2025, and 0.20 mIU/L in May 2025). A TSH-stimulated diagnostic whole-body scan with 5.06 mCi of iodine-131 in January 2025 (stimulated thyroglobulin 7.34 µg/L; TSH 134 mIU/L) showed that the thyroid-bed uptake seen after ablation had resolved and found no iodine-avid lesions elsewhere, a pattern consistent with an incomplete biochemical response. Whether the disease had lost the ability to concentrate iodine or had never had it could not be determined. FDG positron emission tomography/computed tomography (PET/CT) in May 2025, obtained for a thyroglobulin of 11.6 µg/L, revealed multiple bilateral pulmonary nodules suspicious for metastases, the largest being a 7 mm right lower lobe nodule with a maximum standardized uptake value (SUVmax) of 8.9, plus a low-grade right hilar focus (SUVmax of 3.8), thought to be nodal or a small bronchial-related nodule. The thyroid bed showed no other hypermetabolic uptake. The scan extended from the skull vertex, but the report noted that intense physiological cerebral uptake prevented reliable assessment of the brain and advised contrast-enhanced magnetic resonance imaging (MRI) if clinically justified. Between May 2024 and May 2025, the thyroglobulin rose from 1.56 µg/L to 11.6 µg/L even as TSH fell, a doubling time of approximately four months. The pulmonary nodules were not biopsied, and their metastatic nature is inferred from their imaging appearance and the rising thyroglobulin.

About two months later, he developed progressive dizziness and headache and came to the emergency department in July 2025. Chest CT on admission again showed the multiple bilateral pulmonary nodules (Figure 1). Brain MRI with and without intravenous contrast the following day showed a mixed cystic and solid left cerebellar lesion, the solid component measuring 2.5 × 2.2 × 2.8 cm and the lesion 3.3 × 3.2 × 3.1 cm overall, with surrounding vasogenic edema, mild intralesional hemorrhage on susceptibility-weighted imaging, and mass effect deviating the brainstem rightward (Figure 2); the radiological differential was a solitary metastasis versus a primary parenchymal neoplasm such as hemangioblastoma. He was started on intravenous dexamethasone and observed in the intensive care unit, and the day after the scan, he underwent a left suboccipital craniotomy, at which the mass was resected without complication.

**Figure 1:**
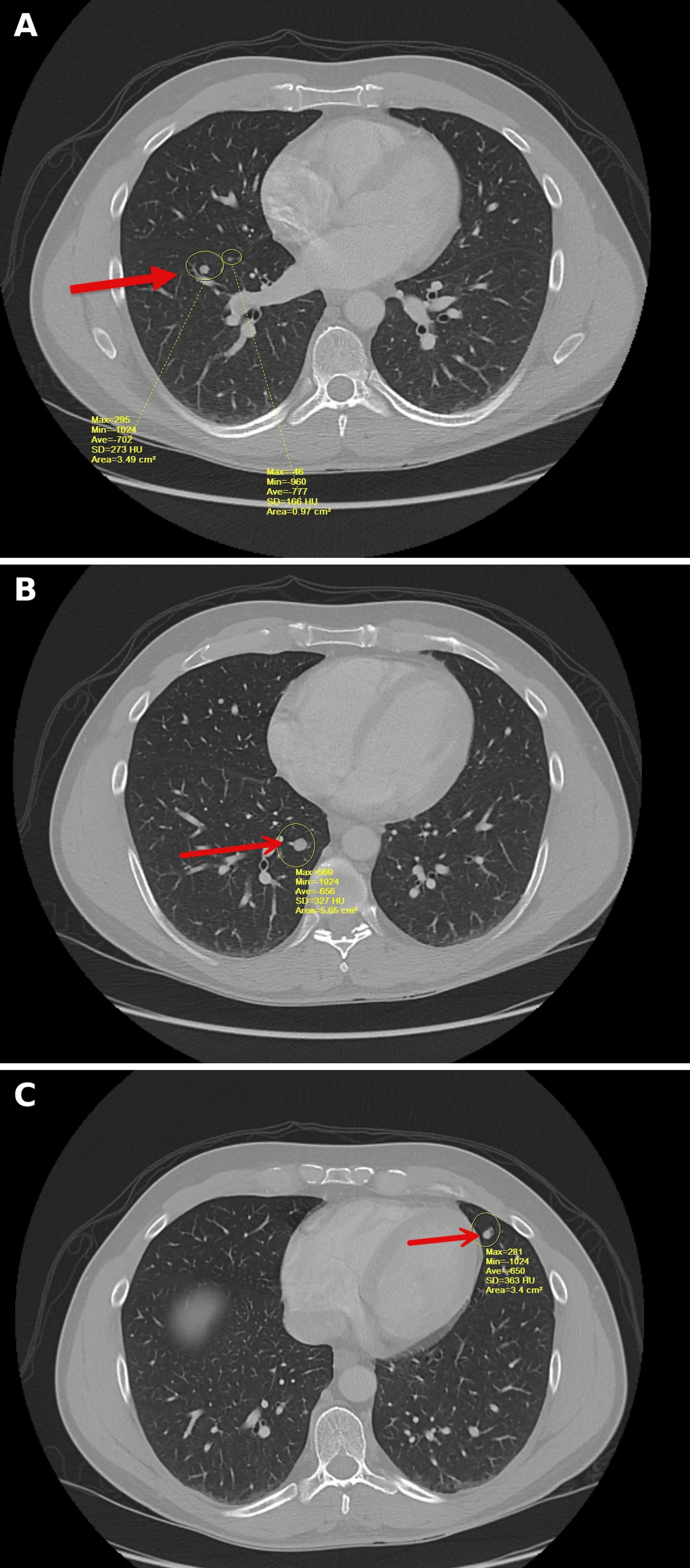
Axial chest computed tomography (CT; lung window) demonstrating bilateral pulmonary nodules suspicious for metastases. (A) The red arrow indicates a right-sided pulmonary nodule; a second smaller adjacent nodule is also circled. (B) The red arrow indicates a right perihilar pulmonary nodule. (C) The red arrow indicates a left-sided pulmonary nodule abutting the cardiac border. Yellow circles and accompanying values are region-of-interest attenuation measurements in Hounsfield units.

**Figure 2:**
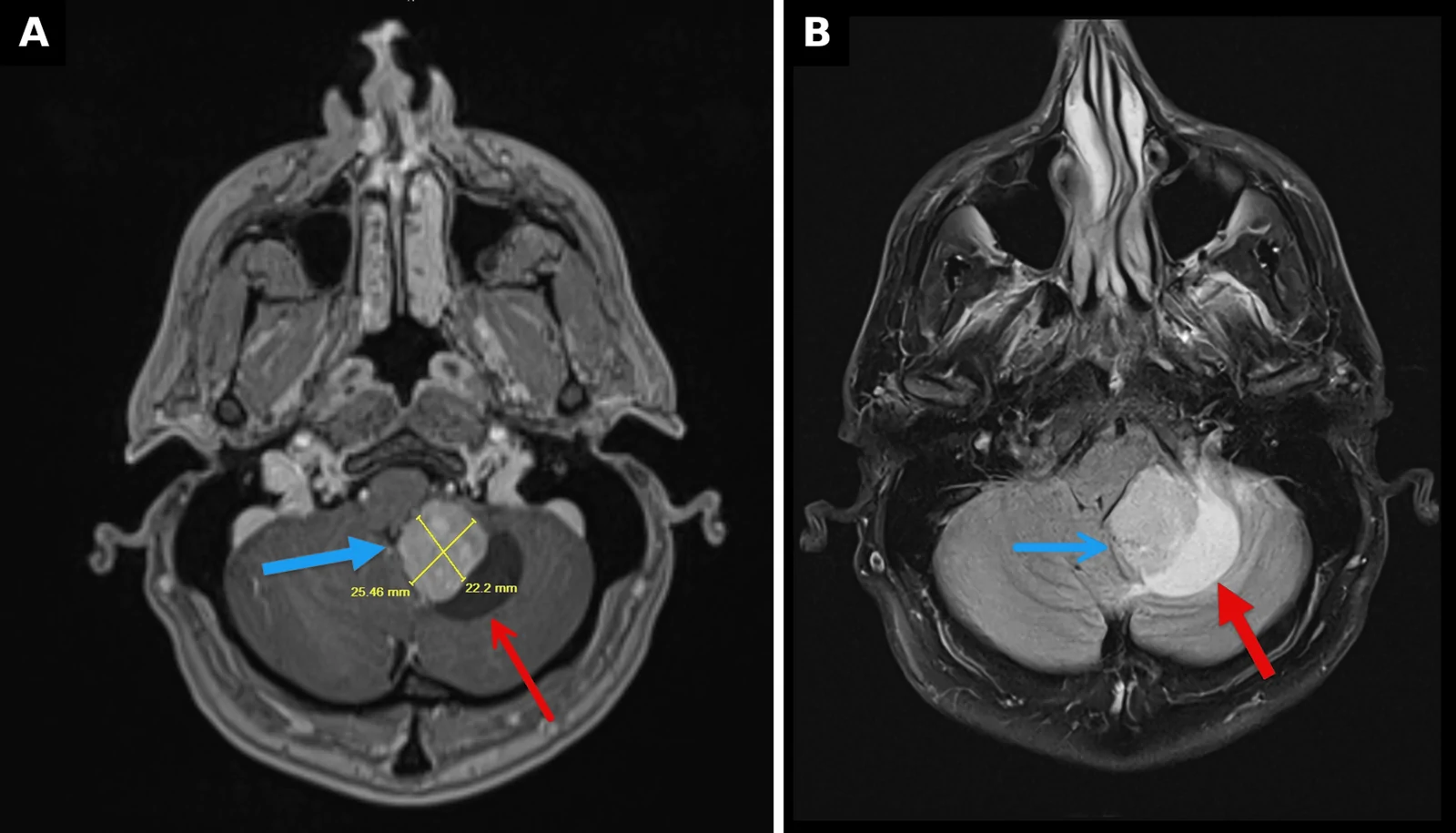
Brain magnetic resonance imaging (MRI) showing the left cerebellar mass with surrounding vasogenic edema. (A) Axial post-contrast T1-weighted (MPRAGE) image: the blue arrow indicates the enhancing solid component of the left cerebellar mass, measuring approximately 25 × 22 mm in this plane (yellow calipers). (B) Axial T2 FLAIR image at the same level: the blue arrow indicates the mass and the red arrow the surrounding hyperintense vasogenic edema. MRI: magnetic resonance imaging; MPRAGE: magnetization-prepared rapid acquisition gradient echo; FLAIR: fluid-attenuated inversion recovery.

The resection specimen, received as a 3.0 × 2.2 × 0.9 cm aggregate of pink-tan to red tissue fragments, contained a malignant epithelioid neoplasm growing in nests and trabeculae. The cells had abundant granular eosinophilic (oncocytic) cytoplasm and round nuclei with prominent nucleoli; occasional multinucleated cells and cells with giant bizarre nuclei were present, and mitoses numbered 8 per 2 mm^2^ in a hotspot, without tumor necrosis (Figure 3). Immunohistochemistry showed diffuse positivity for multicytokeratin AE1/AE3 and CK7 (Figure 4), nuclear TTF-1 (Figure 5), and nuclear PAX-8 (Figure 6), with negative CK20, CDX-2, synaptophysin, chromogranin, and S100. Coexpression of TTF-1 and PAX-8 in a tumor with oncocytic morphology matching the thyroid resection established the diagnosis of metastatic oncocytic thyroid carcinoma. On the basis of mitotic count, the metastasis met WHO 2022 criteria for differentiated high-grade carcinoma with oncocytic features [1]. No papillary carcinoma component was identified in the metastasis.

**Figure 3:**
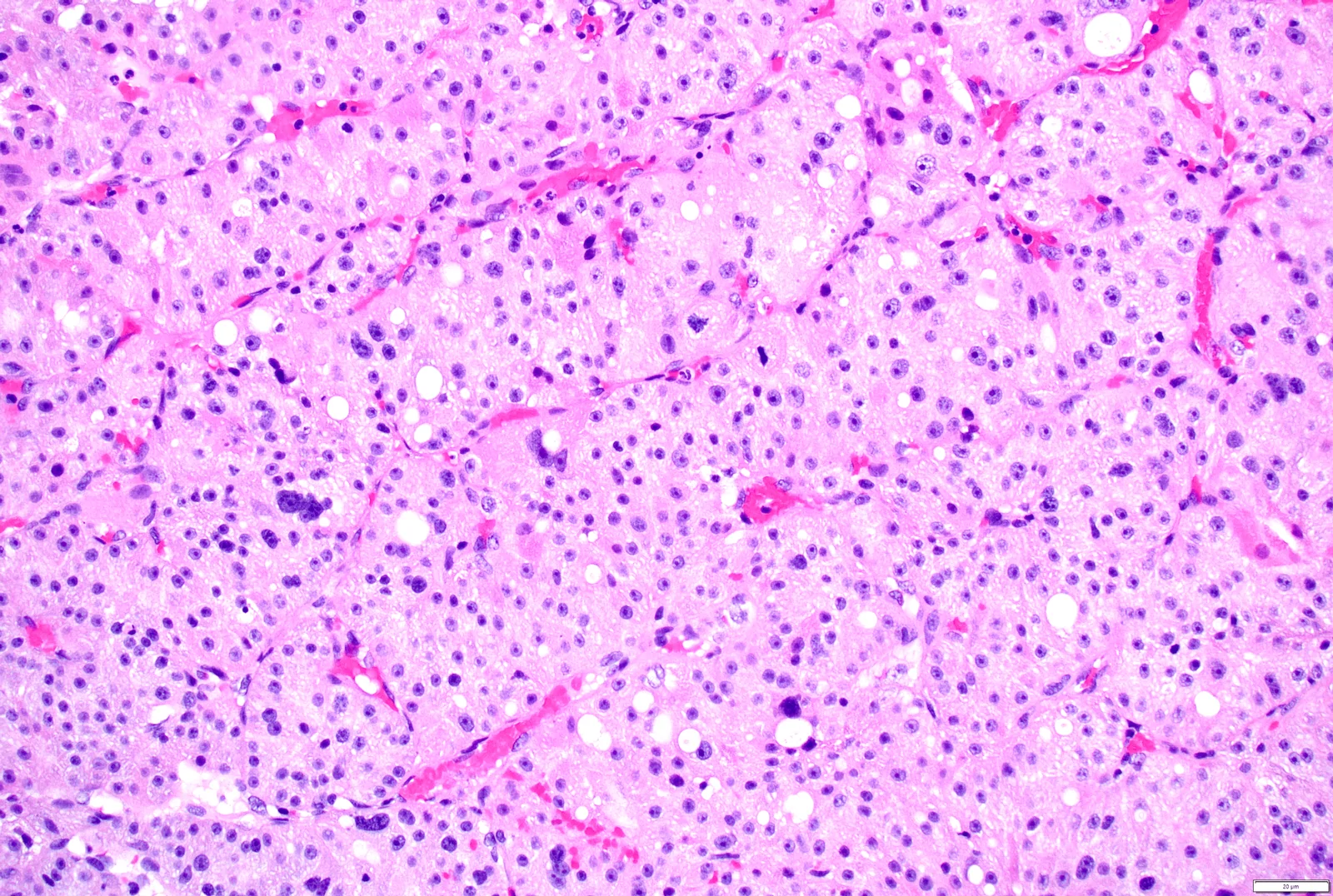
Cerebellar metastasis (hematoxylin and eosin stain: 200×). Malignant epithelioid neoplasm with nested and trabecular growth. Tumor cells show abundant granular eosinophilic (oncocytic) cytoplasm and round nuclei with prominent nucleoli; occasional cells with enlarged, bizarre nuclei are present.

**Figure 4:**
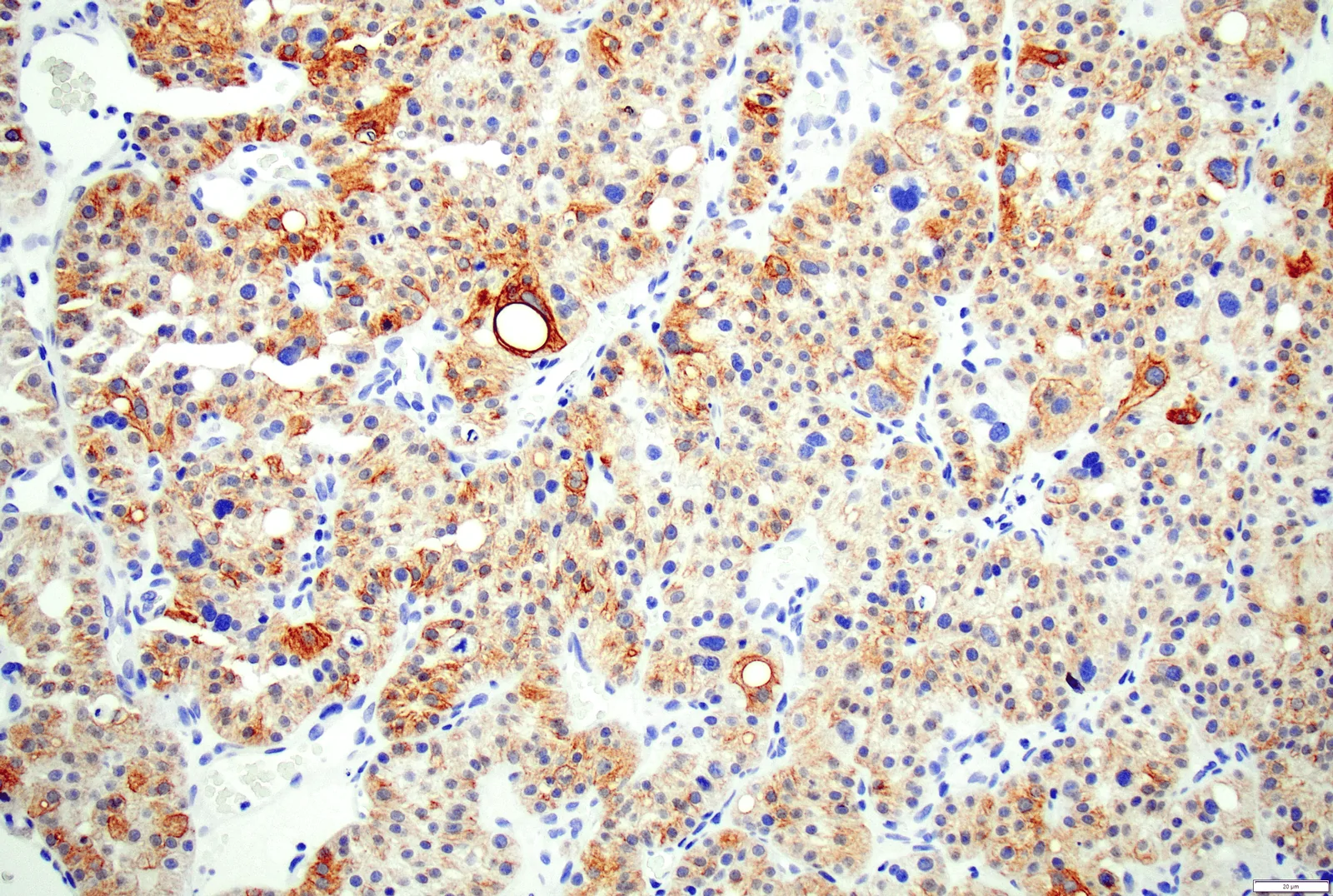
CK7 immunohistochemical stain (200×). Diffuse cytoplasmic and membranous positivity for cytokeratin 7 in the tumor cells.

**Figure 5:**
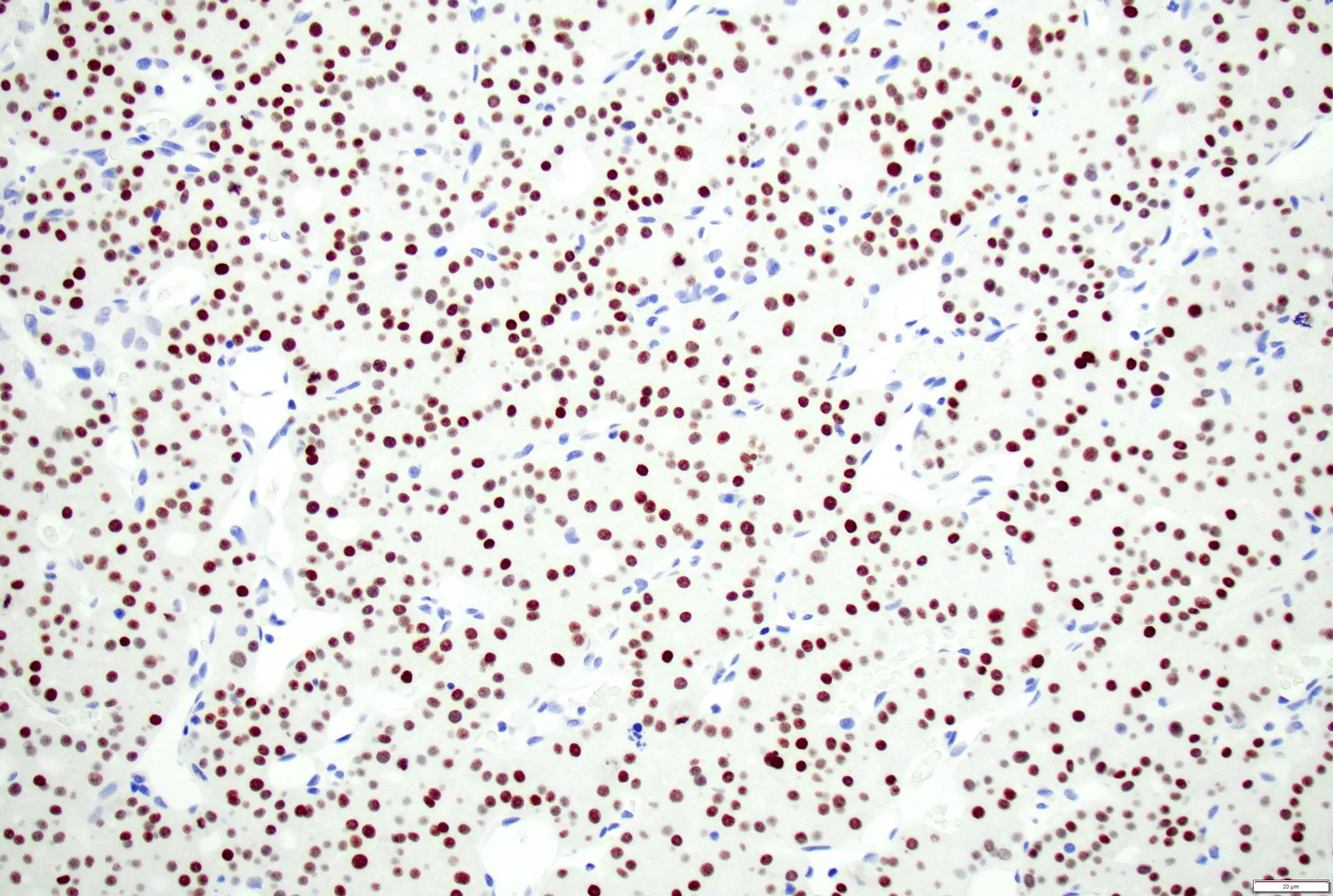
Thyroid transcription factor 1 (TTF-1) immunohistochemical stain (200×). Diffuse nuclear positivity for TTF-1 in the tumor cells. TTF-1: thyroid transcription factor 1.

**Figure 6:**
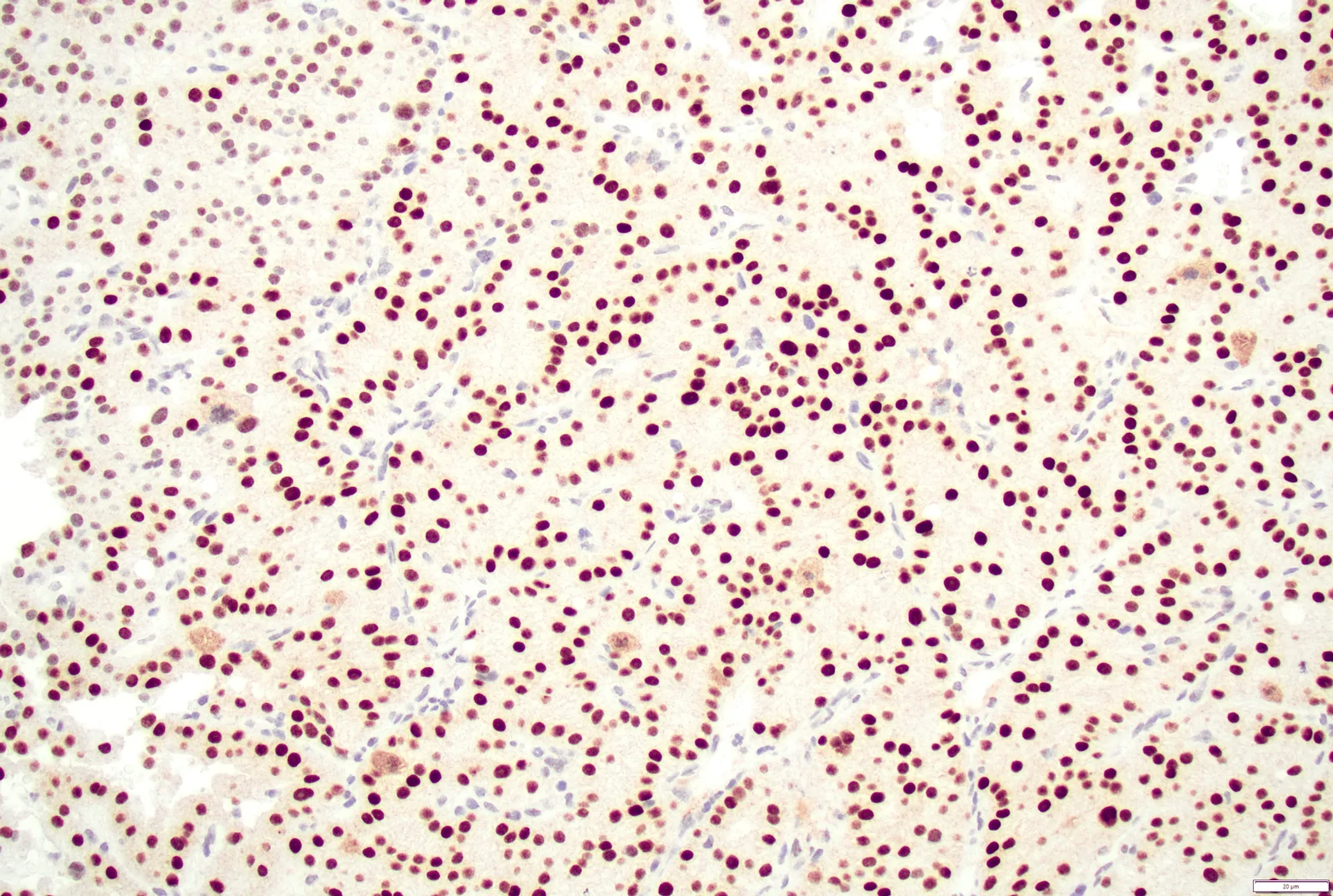
PAX-8 immunohistochemical stain (200×). Strong, diffuse nuclear PAX-8 expression. Together with TTF-1 positivity, this supports the thyroid follicular cell origin of the metastasis.

He recovered without incident, and his neurological symptoms resolved. Stereotactic radiosurgery to the resection cavity was recommended. The patient lives overseas for work and chose to have this treatment abroad; the details of treatment are not available, and he was not seen at our institution after recovery from craniotomy. 

## Discussion

Our patient's course represents, in compressed form, the natural history of aggressive oncocytic carcinoma: a large, widely invasive primary with extensive vascular invasion, negative radioiodine imaging within about a year of ablation, pulmonary nodules suspicious for hematogenous spread, and finally a symptomatic, histologically confirmed brain metastasis, all within two years of diagnosis and before the age of 47. The 2022 World Health Organization classification separates oncocytic carcinoma from follicular carcinoma precisely because their clinical behavior and biology differ [1]. Whole-exome and mitochondrial sequencing studies have found recurrent, often homoplasmic, disruptive mutations in mitochondrial complex I genes, together with loss of heterozygosity so extensive that many tumors approach genome-wide haploidy; these alterations are retained in metastases and offer a plausible mechanistic basis for both radioiodine resistance and avid FDG uptake seen in metastatic disease [2,3].

Whether RAI helps in oncocytic carcinoma is still debated. Uptake is less reliable than in papillary or conventional follicular carcinoma, yet registry data associate adjuvant RAI with better survival in higher-risk patients [9]. Radioiodine is curative mainly in iodine-avid metastatic disease and offers little when metastases do not concentrate iodine [10]. In our patient, the post-therapy scan after 150 mCi showed thyroid-bed uptake only, and the later diagnostic scan, obtained because the thyroglobulin was climbing, was negative despite disease that soon proved structurally evident. A rising thyroglobulin with negative iodine imaging is a biochemical incomplete response rather than proven radioiodine-refractory disease, and it should prompt FDG-PET/CT, which localizes non-iodine-avid recurrence in most patients with an elevated thyroglobulin and a negative iodine whole-body scan [11,12]. The reciprocal relationship between iodine uptake and glucose metabolism, the so-called flip-flop phenomenon, is especially marked in oncocytic tumors, whose mitochondria-laden cells with defective oxidative phosphorylation take up FDG avidly [2,12]. In our patient, whether the tumor lost the ability to concentrate iodine or never had it cannot be determined. 

The earliest warning here was biochemical, not radiological. After an initial postoperative fall, the thyroglobulin rose across four consecutive measurements before anything was visible on imaging, which fits guideline advice that the trend, not any single value, should drive escalation [11]. His doubling time of about four months was short, and the rise continued after TSH had been suppressed; a doubling time of less than one year is associated with poorer outcomes [11]. The FDG-PET/CT performed two months before he became symptomatic included the brain, but its report stated that intense physiological cerebral uptake prevented reliable assessment and suggested contrast-enhanced MRI if clinically justified; brain imaging was not obtained until symptoms developed. Because physiological glucose uptake limits PET for intracranial metastases, contrast-enhanced MRI remains the study of choice for the brain in patients with metastatic thyroid cancer.

Brain metastases complicate roughly 1% of differentiated thyroid cancers, usually in older patients with bulky metastatic burden, and shorten survival considerably, though resection has repeatedly been associated with longer survival in suitable candidates [4]. Contemporary practice combines resection or stereotactic radiosurgery with TSH suppression and, when needed, systemic therapy, reserving whole-brain radiotherapy for diffuse disease; solitary accessible lesions such as this one achieve the best outcomes [5]. Resection gave our patient immediate relief of posterior fossa symptoms and a tissue diagnosis in one step, and adjuvant stereotactic radiosurgery to the cavity was recommended afterward, in line with current guidance [5,11]. Should the suspected pulmonary metastases progress and prove radioiodine-refractory, molecular profiling and a multikinase inhibitor such as lenvatinib, which prolongs progression-free survival in radioiodine-refractory disease, would be the logical next step [13].

Confirming that an intracranial epithelioid tumor is thyroid in origin rests on a focused immunohistochemical panel. Diffuse TTF-1 and PAX-8 coexpression with CK7 positivity, against negative CK20, CDX-2, synaptophysin, chromogranin, and S100, excluded the main alternatives: lung adenocarcinoma (typically TTF-1 positive but PAX-8 negative), renal cell carcinoma (PAX-8 positive but TTF-1 negative), neuroendocrine tumors, melanoma, and primary posterior fossa lesions such as hemangioblastoma. PAX-8 is retained across the spectrum of thyroid epithelial neoplasms, and, paired with TTF-1, it is highly specific for a thyroid primary in a metastatic site [8,14]. The oncocytic cytomorphology, mirroring the thyroid resection, settled the matter. With 8 mitoses per 2 mm^2^, the metastasis met the WHO 2022 criteria for differentiated high-grade thyroid carcinoma, a category associated with worse outcomes than well-differentiated thyroid carcinoma [1]. The primary could not be graded on review, although its report described focal tumor necrosis, which would also meet this definition. 

The patient's age deserves comment. Oncocytic carcinoma is chiefly a disease of the sixth and seventh decades, and brain metastases from thyroid cancer likewise cluster in older patients [1,4]. Because he was younger than 55, the AJCC system placed him at stage I at diagnosis and would place him only at stage II with distant spread, which understates how aggressive his disease proved to be [8]. That he developed widely invasive disease with negative radioiodine imaging within about a year of treatment and spread to the cerebellum at age 46 shows that a favorable age-based stage offers no protection when the histology is high risk (wide invasion and extensive vascular invasion above all) and argues for close biochemical and cross-sectional surveillance in such patients.

This report has limitations. The pulmonary nodules were not confirmed histologically. The patient continued his care overseas, so the delivery of stereotactic radiosurgery could not be documented, and no follow-up after recovery from craniotomy is available. Local control of the cerebellar metastasis and the course of the pulmonary disease therefore cannot be judged from this case. The primary tumor slides were not available for review, and no mitotic count was reported for the primary. Although suppression is advised for high-risk disease [11], his TSH was not suppressed during the first eight months of levothyroxine therapy (2.01 and 3.96 mIU/L); later values were 0.01-0.29 mIU/L, and the reason for the initial level is not documented. Whether this contributed to progression is unknown, although thyroglobulin continued to rise after TSH was suppressed.

## Conclusions

Widely invasive oncocytic (Hurthle cell) carcinoma can behave aggressively even in patients younger than 55, and a thyroglobulin that keeps rising despite negative iodine scintigraphy is a biochemical incomplete response that should prompt a search for structural disease, usually with FDG PET. That study cannot reliably exclude brain metastases, as its report in our patient noted, and new neurological symptoms call for dedicated brain MRI without delay.

For a solitary, accessible cerebellar metastasis, resection is a reasonable option. In our patient, it relieved symptoms and provided the tissue on which the diagnosis rests, with coexpression of TTF-1 and PAX-8 confirming the thyroid origin. Adjuvant stereotactic radiosurgery was recommended, but follow-up was not available, so local control could not be assessed. In our patient, the brain lesion was found because of symptoms, not because of the thyroglobulin trend. Brain MRI may be worth considering in patients with radioiodine-refractory distant metastases, particularly those with oncocytic carcinoma, so that intracranial disease can be found before it causes symptoms. 
